# Publisher Correction: Several multiple sequence alignment-perturbing methods enhance AlphaFold3 sampling of alternative protein states

**DOI:** 10.1038/s42004-026-02247-5

**Published:** 2026-10-09

**Authors:** Samuel Eriksson Lidbrink, Ivan Nissen, Jonathan Kenichi Ahrlind, Rebecca J. Howard, Erik Lindahl

**Affiliations:** 1 Science for Life Laboratory, Department of Biochemistry and Biophysics, Stockholm University, Solna, Sweden; 2 Science for Life Laboratory, Department of Applied Physics, KTH Royal Institute of Technology, Solna, Sweden; 3 Department of Physics, Chemistry and Biology, Linköping University, Linköping, Sweden; 4 Department of Chemistry, University of Illinois at Urbana-Champaign, Urbana, IL USA

**Keywords:** Molecular modelling, Proteins

Correction to: *Communications Chemistry* https://doi.org/10.1038/s42004-026-02198-x, published online 18 September 2026

In this article, the order in which the authors appeared in the author list was incorrectly given as Samuel Eriksson Lidbrink, Ivan Nissen, Rebecca J. Howard, Jonathan Kenichi Ahrlind, Erik Lindahl, where it should have been Samuel Eriksson Lidbrink, Ivan Nissen, Jonathan Kenichi Ahrlind, Rebecca J Howard, Erik Lindahl.

