# Supplementary material for: Several multiple sequence alignment-perturbing methods enhance AlphaFold3 sampling of alternative protein states
Source: Commun Chem. 2026 Sep 18;9:300. doi: 10.1038/s42004-026-02198-x (PMC13588791; doi:10.1038/s42004-026-02198-x)
Supplement: Supplementary file 1 [file 42004_2026_2198_MOESM1_ESM.pdf]

nature portfolio

Peer Review File

Several multiple sequence alignment-perturbing methods enhance AlphaFold3 sampling of alternative protein states

Corresponding Author: Dr Rebecca Howard

This file contains all reviewer reports in order by version, followed by all author rebuttals in order by version.

Version 0:

Reviewer comments:

Reviewer #1

(Remarks to the Author)
The paper makes an interesting and non-obvious observation. By masking out regions in the MSA, it is possible to coerce AlphaFold to generate alternative conformations for a protein that naturally has multiple configurations. This is an interesting finding overall, and particularly timely as multiple configurations have been AlphaFold's Achillies heel. There are comparisons made to other approaches to this problem, such as BioEmu. It is notable that the additional conformational diversity does not lead to better results than AF3 on its own. The manuscript highlights that F-masking shows some improvement over the typical X-masking, which is an interesting finding in its own right. I have suggestions, but think that this paper is effectively ready for publication.

How much do you think it matters that the new protein structures released after the training cutoff are likely super similar to proteins that *are* in the training database? A fun exercise I have done while teaching AlphaFold to students is to set the cutoff date at the Unix epoch, where there are basically no structures. AlphaFold2 and 3 are surprisingly effective, as the model weights and the MSAs are still enough to guide the structure.

The Zenodo archive is good enough to reproduce the results, although I personally would have preferred github due to the nature of the code and results presented.

Reviewer #2

(Remarks to the Author)
I co-reviewed this manuscript with one of the reviewers who provided the listed reports. This is part of the Communications Chemistry initiative to facilitate training in peer review and to provide appropriate recognition for Early Career Researchers who co-review manuscripts.

Reviewer #3

(Remarks to the Author)
The present manuscript investigates the ability of AlphaFold3 (AF3) to sample alternative conformational states and explores the use of MSA perturbation strategies (column masking, clustering, and stochastic subsampling) to enhance this capability.

The topic is timely and of broad interest to the structural biology and computational modeling communities. The results suggest that AF3 improves over AF2 in sampling alternative conformations and that simple modifications of the MSA can further enhance this behavior. Overall, I find the work interesting and potentially impactful, and I would support publication. However, several aspects of the analysis and interpretation require clarification and strengthening before the conclusions can be fully supported. I therefore recommend publication after revisions.

The main claims of the manuscript are that:
(i) AF3 samples alternative conformations more effectively than AF2,
(ii) MSA perturbation strategies significantly enhance the sampling of alternative states,

(iii) these improvements occur even when the latent space is fixed (i.e., under a fixed random seed), suggesting that multiple conformations may be encoded within a single latent representation.

These claims are relevant and, in particular, the idea that AF3 may encode multiple conformations within a single latent space could influence how AlphaFold predictions are interpreted beyond static structures. The work builds on previous studies (e.g., AFsample-type approaches), but extends them to AF3 and provides a broader benchmarking, which constitutes a useful contribution.

1. The analysis relies heavily on the top 1% TM-score metric, which emphasizes best-case predictions. While informative, this metric may overestimate performance and does not capture the overall sampling distribution. Reporting additional statistics (e.g., median TM-score, distribution of scores, or probability of sampling a correct conformation) would provide a more complete and robust evaluation.

2. The impact of MSA perturbations on prediction confidence is not evaluated. In particular, metrics such as pLDDT or PAE are not reported. This is an important omission, as reducing MSA constraints is expected to increase uncertainty. Without these metrics, it is difficult to distinguish between meaningful alternative conformations and artifacts arising from reduced evolutionary information.

3. The conclusion that multiple conformations are sampled from a single latent space is based on indirect evidence (fixed-seed experiments). While suggestive, this interpretation is not directly supported by analysis of model representations. Additional analyses (e.g., clustering per seed or quantifying structural diversity within each seed) would strengthen this claim.

4. The observed dependence on the choice of masking amino acid (e.g., phenylalanine versus X) is intriguing but lacks mechanistic explanation. It remains unclear whether this effect reflects meaningful perturbations of sequence constraints or introduces biases that favor specific conformations. Further discussion or analysis would strengthen this point.

5. The manuscript would benefit from a clearer indication of the practical computational cost associated with implementing the proposed sampling strategies. While the study explores extensive sampling (e.g., ~1000 predictions per protein), it is unclear how this translates to a realistic use case for an individual researcher. It would be helpful if the authors could provide a concrete example of a typical workflow, including an approximate number of required samples and a rough estimate of computational cost (e.g., GPU time) for a single protein. Such clarification would significantly improve the accessibility and practical applicability of the proposed approach.

In summary, this manuscript presents an interesting and relevant contribution to the study of conformational sampling in AF3. The results are promising, but several aspects of the analysis and interpretation would benefit from further clarification and quantitative support. Addressing the points above would significantly strengthen the manuscript and improve its impact.

Reviewer #4

(Remarks to the Author)
This manuscript evaluates several MSA perturbation strategies previously shown to enhance conformational sampling in AlphaFold2 (AF2), and assesses their impact on AlphaFold3 (AF3). Using a benchmark of 107 proteins with multiple experimentally determined conformations, the authors report that unperturbed AF3 already recovers alternative states with significantly higher TM-scores than AF2 and at a level comparable to BioEmu. They further show that all tested MSA perturbation strategies yield statistically significant improvements in AF3 sampling, with limited risk of degrading performance.

Overall, the study is relevant and the benchmark is useful. However, the contribution appears mostly incremental with respect to existing work (e.g., AFsample-like strategies), and the manuscript remains largely descriptive. A stronger mechanistic interpretation is needed to justify the broader significance of the results.

A major point that deserves further investigation concerns the role of AF3's diffusion model. Since diffusion-based generation is a key architectural change between AF2 and AF3, it is important to clarify to what extent the improved sampling of AF3 originates from diffusion itself. In this context, it is surprising that the authors focus exclusively on MSA perturbations while not exploring diffusion sampling parameters. Adjusting diffusion-related inference settings could potentially increase structural diversity in a more direct and principled way than MSA manipulation. Even if such parameters are not fully exposed in the public inference pipeline, the authors should discuss point more deeply and, if possible, include experiments probing whether diffusion sampling controls can modulate the exploration of alternative conformations.

Additionally, the manuscript would benefit from explicit diversity analyses. The reported improvements are mainly based on top 1% TM-score, which does not fully characterize sampling behavior. The authors should quantify and visualize the diversity of generated ensembles, and assess whether perturbation methods broaden conformational space or actually bias predictions toward a subset of known structures. Relatedly, the tradeoff between diversity and plausibility should be evaluated more carefully, since increased diversity may also produce unrealistic conformations.
The comparison with BioEmu should also be framed more cautiously. BioEmu is designed to sample a Boltzmann distribution, whereas AlphaFold models do not explicitly aim to reproduce thermodynamic ensembles. Therefore, direct comparison on the task of recovering experimental alternative conformations may not be fully fair without clarifying what is being measured: recall of known states versus sampling of physically meaningful distributions.

Finally, the dataset requires clearer description: are alternative conformations observed under comparable experimental conditions, or are they driven by binding partners, ligands, or environmental changes (pH, ionic strength, etc.) that are not represented in the AF3 inputs? This could strongly affect interpretation, and might explain cases where BioEmu (and also AF) fails to sample certain states that are very unlikely in the absence of an interacting molecule, for instance.

Version 1:

Reviewer comments:

Reviewer #1

(Remarks to the Author)
I think the authors have done a good job of responding to the points raised by the reviewers, and I recommend publication.

Reviewer #2

(Remarks to the Author)
I co-reviewed this manuscript with one of the reviewers who provided the listed reports. This is part of the Communications Chemistry initiative to facilitate training in peer review and to provide appropriate recognition for Early Career Researchers who co-review manuscripts.

Reviewer #3

(Remarks to the Author)
The authors have adequately addressed the concerns raised. The revisions and clarifications provided have significantly improved the clarity of the manuscript, better defined its scope, and strengthened the presentation and impact of the work.

Reviewer #4

(Remarks to the Author)
I thank the authors for their careful revision of the manuscript and for providing detailed responses to the reviewers' comments. I appreciate the substantial effort invested in improving the work. In particular, the additional analyses of sampling diversity and the expanded discussion have strengthened the manuscript.

That said, my main concerns are only partially addressed.

My original review emphasized that the study is primarily descriptive and that its scientific impact would be significantly enhanced by a stronger mechanistic understanding of why the proposed perturbation strategies improve conformational sampling in AF3. The additional discussion regarding the effects of amino acid masking is welcome, but it remains largely speculative. Likewise, the discussion of the diffusion model highlights interesting future directions but does not substantially clarify the origin of the observed improvements.

Consequently, I continue to view this work as an incremental extension of previous studies on MSA perturbation strategies rather than a fundamentally new methodological advance. Nevertheless, I acknowledge that the benchmark is extensive, the analyses are carefully conducted, and the results should be valuable to researchers interested in AF3 conformational sampling.

Open Access This Peer Review File is licensed under a Creative Commons Attribution 4.0 International License, which permits use, sharing, adaptation, distribution and reproduction in any medium or format, as long as you give appropriate credit to the original author(s) and the source, provide a link to the Creative Commons license, and indicate if changes were made.
In cases where reviewers are anonymous, credit should be given to 'Anonymous Referee' and the source.
The images or other third party material in this Peer Review File are included in the article's Creative Commons license, unless indicated otherwise in a credit line to the material. If material is not included in the article's Creative Commons license and your intended use is not permitted by statutory regulation or exceeds the permitted use, you will need to obtain permission directly from the copyright holder.
To view a copy of this license, visit https://creativecommons.org/licenses/by/4.0/

Thank you for the thoughtful consideration of our manuscript (COMMSCHEM-26-0288). As demonstrated in the attached revision and outlined below, we believe we have responded to all concerns of reviewers 1–4, including edits to the Methods, Results, Discussion and References, and addition of Supplementary Figures 1, 3, 4, 6 and 7.

During the revision, we also noted that one out of the 107 proteins we analyzed had been predicted as a dimer, instead of a monomer as intended. We have corrected this, resulting in marginal updates to Figure 2 and Supplementary Figures 2, 5 and 8 (previously numbered Supplementary Figures 1, 2 and 3) that do not materially alter any inferences or conclusions of the manuscript.

In this response, reviewer comments are gray (renumbered for clarity) and quotes from the revised manuscript are red.

Reviewer #1: The paper makes an interesting and non-obvious observation. By masking out regions in the MSA, it is possible to coerce AlphaFold to generate alternative conformations for a protein that naturally has multiple configurations. This is an interesting finding overall, and particularly timely as multiple configurations have been AlphaFold's Achillies heel. There are comparisons made to other approaches to this problem, such as BioEmu. It is notable that the additional conformational diversity does not lead to better results than AF3 on its own. The manuscript highlights that F-masking shows some improvement over the typical X-masking, which is an interesting finding in its own right. I have suggestions, but think that this paper is effectively ready for publication.

1.1. How much do you think it matters that the new protein structures released after the training cutoff are likely super similar to proteins that *are* in the training database? A fun exercise I have done while teaching AlphaFold to students is to set the cutoff date at the Unix epoch, where there are basically no structures. AlphaFold2 and 3 are surprisingly effective, as the model weights and the MSAs are still enough to guide the structure.

Response 1.1. We thank the reviewer for noting the quality and timeliness of the work. We agree that AlphaFold displays a remarkable ability to generalize to unseen cases, and now elaborate on this in the Discussion (p. 13):

Here, we have shown that AF3 significantly improves the sampling of alternative states over AF2 on a dataset with over 100 different proteins, that AF3 performs comparably to BioEmu for the alternative states and better for the preferred states, and that MSA perturbations and particularly column masking improve AF3 sampling further. This observation extends to proteins where all experimental structures have been released after the AF3 training cutoff, showcasing the generalizability to unseen cases.

1.2. The Zenodo archive is good enough to reproduce the results, although I personally would have preferred github due to the nature of the code and results presented.

Response 1.2. We appreciate the recognition of our effort to make our results reproducible. Although github is also an important tool for our group, we typically deposit code and data associated with publications on Zenodo to keep a static permanent record. We have now updated the deposition with additional data for the figures added or modified in revision.

Reviewer #2: I co-reviewed this manuscript with one of the reviewers who provided the listed reports. This is part of the Communications Chemistry initiative to facilitate training in peer review and to provide appropriate recognition for Early Career Researchers who co-review manuscripts.

Response 2. We thank the reviewer for their time and attention.

Reviewer #3: The present manuscript investigates the ability of AlphaFold3 (AF3) to sample alternative conformational states and explores the use of MSA perturbation strategies (column masking, clustering, and stochastic subsampling) to enhance this capability.

The topic is timely and of broad interest to the structural biology and computational modeling communities. The results suggest that AF3 improves over AF2 in sampling alternative conformations and that simple modifications of the MSA can further enhance this behavior. Overall, I find the work interesting and potentially impactful, and I would support publication. However, several aspects of the analysis and interpretation require clarification and strengthening before the conclusions can be fully supported. I therefore recommend publication after revisions.

The main claims of the manuscript are that:

(i) AF3 samples alternative conformations more effectively than AF2,

(ii) MSA perturbation strategies significantly enhance the sampling of alternative states,

(iii) these improvements occur even when the latent space is fixed (i.e., under a fixed random seed), suggesting that multiple conformations may be encoded within a single latent representation.

These claims are relevant and, in particular, the idea that AF3 may encode multiple conformations within a single latent space could influence how AlphaFold predictions are interpreted beyond static structures. The work builds on previous studies (e.g., AFsample-type approaches), but extends them to AF3 and provides a broader benchmarking, which constitutes a useful contribution.

3.1. The analysis relies heavily on the top 1% TM-score metric, which emphasizes best-case predictions. While informative, this metric may overestimate performance and does not capture the overall sampling distribution. Reporting additional statistics (e.g., median TM-score,

distribution of scores, or probability of sampling a correct conformation) would provide a more complete and robust evaluation.

Response 3.1. We thank the reviewer for noting the relevance and timeliness of the work. Whereas we focused our original study on best-case predictions, we agree on the utility of overall statistics. To this end, we now highlight the probabilities of sampling each state in new Supplementary Figure 7, with accompanying description in Results (p. 7):

For more modest amounts of sampling, we found that unperturbed AF3 generally sampled a larger fraction of predictions closely resembling the alternative state compared to both BioEmu and AF2, and that MSA perturbations and especially column masking further increased this sampling (Supplementary Figure 7). For instance, out of the 107 investigated proteins, there were 32 and 27 for which BioEmu and AF2 respectively sampled at least 20% of predictions with TM-scores above 0.9 compared to the alternative state; unperturbed AF3 did so for 35 cases, and AF3 with column masking, clustering and stochastic subsampling did so for 48, 45, and 43 cases, respectively.

3.2. The impact of MSA perturbations on prediction confidence is not evaluated. In particular, metrics such as pLDDT or PAE are not reported. This is an important omission, as reducing MSA constraints is expected to increase uncertainty. Without these metrics, it is difficult to distinguish between meaningful alternative conformations and artifacts arising from reduced evolutionary information.

Response 3.2. We regret our earlier omission of the confidence scores, and now report these values in the new Supplementary Figure 3, and have updated the Results (p. 4):

Overall, we found that both the alternative and preferred states were sampled with significantly higher top 1% TM-scores in unperturbed AF3 compared to AF2 (using the Wilcoxon signed-rank test for statistical significance) (Figure 2A, Supplementary Figure 2A). MSA column masking, clustering and stochastic subsampling all significantly increased these scores for the alternative states (Figure 2A), although overall prediction confidence modestly decreased based on pTM, average pLDDT and PAE scores, as expected with a decrease in evolutionary information (Supplementary Figure 3).

3.3. The conclusion that multiple conformations are sampled from a single latent space is based on indirect evidence (fixed-seed experiments). While suggestive, this interpretation is not directly supported by analysis of model representations. Additional analyses (e.g., clustering per seed or quantifying structural diversity within each seed) would strengthen this claim.

Response 3.3. We agree with the reviewer that it is an important point whether the improvements are present for fixed latent spaces, and that additional analyses can elucidate this point further. We now also quantify the diversity of fixed latent spaces using the Vendi score in the new Supplementary Figure 4, which provides further evidence that the perturbation techniques indeed enhance the diversity of the latent spaces. As also quoted in Response 4.3, we have updated the Results accordingly (p. 4):

We saw a similar improvement when we compared the alternative states per fixed latent space in AF3, i.e. a given random seed per protein and model (Supplementary Figure 1B). To further elucidate this point, we also computed the pairwise TM-score similarities of all latent space predictions using Foldseek [20] and quantified the diversity of the latent spaces using Vendi score, a metric representing the number of dissimilar predictions [21]. All perturbation methods significantly increased Vendi scores (Supplementary Figure 4), even when filtering out predictions with MolProbity scores above 3 to mitigate effects from misfolded predictions [22]. These results indicate that the perturbation methods improve AF3's ability to generate a single latent space from which both experimental states can be sampled, and do not require different states to be sampled from distinct latent spaces.

3.4. The observed dependence on the choice of masking amino acid (e.g., phenylalanine versus X) is intriguing but lacks mechanistic explanation. It remains unclear whether this effect reflects meaningful perturbations of sequence constraints or introduces biases that favor specific conformations. Further discussion or analysis would strengthen this point.

Response 3.4. We agree that the dependence on the choice of amino acid mask is intriguing. As also noted in Response 4.1, we have now expanded on the mechanistic possibilities in Discussion (p. 13):

We cannot exclude the possibility that the improved sampling upon change of amino acid column mask may arise at least in part from overfitting to training data. However, since the switch of amino acid mask also improved the top 1% TM score even when the experimental reference was released after the AF3 training date cutoff (Supplementary Figure 8), it is unlikely to fully account for the improvement. Whereas X signifies a lack of information for a given residue, columns masked by an amino acid mask such as F could act as conserved residues of the given type. AF3 may have learned to place these residues in specific environments, since conserved residues may cluster at specific sites in proteins [28-30].

3.5. The manuscript would benefit from a clearer indication of the practical computational cost associated with implementing the proposed sampling strategies. While the study explores extensive sampling (e.g., ~1000 predictions per protein), it is unclear how this translates to a realistic use case for an individual researcher. It would be helpful if the authors could provide a concrete example of a typical workflow, including an approximate number of required samples and a rough estimate of computational cost (e.g., GPU time) for a single protein. Such clarification would significantly improve the accessibility and practical applicability of the proposed approach.

Response 3.5. We appreciate that these practical considerations are useful for the applicability of the methods. We have added the new Supplementary Figure 1 to highlight the computational cost of sampling 1000 AF3 predictions, and have updated the Results accordingly (p. 4):

For each protein and method, at least 1000 protein structures were generated. The computational cost of sampling 1000 AF3 predictions depended on the size of the protein, e.g. taking ~0.5 h, ~3 h, and ~10 h for proteins with $\lesssim$250, ~500-800, and ~1300-1400 residues,

respectively (excluding MSA generation), on a single NVIDIA A100-SXM4-40GB GPU (Supplementary Figure 1).

We also examine the performance of the methods in the new Supplementary Figure 6 and comment on the dependence of sampling quality on the number of samples (p. 7)

For practical applications, it is relevant to comment on the amount of sampling required to obtain satisfactory results. Generally, we found that the best TM-score, especially for the alternative state, increased with the number of sampled predictions, but the extent varied by method (Supplementary Figure 6). Whereas the best mean score for the alternative state largely plateaued after tens of predictions for AF2, the score increased until at least a few hundred predictions were sampled with both perturbed and unperturbed AF3, and rose sharply for BioEmu even when sampling 1000 predictions.

In summary, this manuscript presents an interesting and relevant contribution to the study of conformational sampling in AF3. The results are promising, but several aspects of the analysis and interpretation would benefit from further clarification and quantitative support. Addressing the points above would significantly strengthen the manuscript and improve its impact.

Reviewer #4: This manuscript evaluates several MSA perturbation strategies previously shown to enhance conformational sampling in AlphaFold2 (AF2), and assesses their impact on AlphaFold3 (AF3). Using a benchmark of 107 proteins with multiple experimentally determined conformations, the authors report that unperturbed AF3 already recovers alternative states with significantly higher TM-scores than AF2 and at a level comparable to BioEmu. They further show that all tested MSA perturbation strategies yield statistically significant improvements in AF3 sampling, with limited risk of degrading performance.

4.1. Overall, the study is relevant and the benchmark is useful. However, the contribution appears mostly incremental with respect to existing work (e.g., AFsample-like strategies), and the manuscript remains largely descriptive. A stronger mechanistic interpretation is needed to justify the broader significance of the results.

Response 4.1. We appreciate this point, also highlighted by reviewer 3. As noted in Response 3.4, the dependence on the choice of amino acid mask is especially intriguing, and we have expanded on the mechanistic possibilities in Discussion (p. 13):

We cannot exclude the possibility that the improved sampling upon change of amino acid column mask may arise at least in part from overfitting to training data. However, since the switch of amino acid mask also improved the top 1% TM score even when the experimental reference was released after the AF3 training date cutoff (Supplementary Figure 8), it is unlikely to fully account for the improvement. Whereas X signifies a lack of information for a given residue, columns masked by an amino acid mask such as F could act as conserved residues of the given type. AF3 may have learned to place these residues in specific environments, since conserved residues may cluster at specific sites in proteins [28-30].

4.2. A major point that deserves further investigation concerns the role of AF3's diffusion model. Since diffusion-based generation is a key architectural change between AF2 and AF3, it is important to clarify to what extent the improved sampling of AF3 originates from diffusion itself. In this context, it is surprising that the authors focus exclusively on MSA perturbations while not exploring diffusion sampling parameters. Adjusting diffusion-related inference settings could potentially increase structural diversity in a more direct and principled way than MSA manipulation. Even if such parameters are not fully exposed in the public inference pipeline, the authors should discuss point more deeply and, if possible, include experiments probing whether diffusion sampling controls can modulate the exploration of alternative conformations.

Response 4.2. We agree with the reviewer that modifications of diffusion-related parameters provide a fascinating avenue for sampling alternative states. We humbly acknowledge that we are unable to incorporate such experiments within the scope of this study, but as suggested by the reviewer, we now elaborate on this point in the Discussion (p.14):

Whereas we have focused here on how MSA perturbations can enhance AF3 sampling of alternative states, modifications to AF3's diffusion module could offer complementary approaches to increase the sampled diversity. For instance, it has been shown that repulsive potentials between samples in the diffusion module can enhance the diversity of the generated ensembles [33, 34]. The utility of combining diffusion-related modifications with MSA perturbations remains a particularly interesting avenue for future studies.

4.3. Additionally, the manuscript would benefit from explicit diversity analyses. The reported improvements are mainly based on top 1% TM-score, which does not fully characterize sampling behavior. The authors should quantify and visualize the diversity of generated ensembles, and assess whether perturbation methods broaden conformational space or actually bias predictions toward a subset of known structures. Relatedly, the tradeoff between diversity and plausibility should be evaluated more carefully, since increased diversity may also produce unrealistic conformations.

Response 4.3. As described in Response 3.3, we now quantify the diversity of the generated ensembles, conditioned on model plausibility, in new Supplementary Figure 4. As also quoted in Response 3.3, we also now comment on this observation in Results (p. 4):

We saw a similar improvement when we compared the alternative states per fixed latent space in AF3, i.e. a given random seed per protein and model (Supplementary Figure 1B). To further elucidate this point, we also computed the pairwise TM-score similarities of all latent space predictions using Foldseek [20] and quantified the diversity of the latent spaces using Vendi score, a metric representing the number of dissimilar predictions [21]. All perturbation methods significantly increased Vendi scores (Supplementary Figure 4), even when filtering out predictions with MolProbity scores above 3 to mitigate effects from misfolded predictions [22]. These results indicate that the perturbation methods improve AF3's ability to generate a single latent space from which both experimental states can be sampled, and do not require different states to be sampled from distinct latent spaces.

4.4. The comparison with BioEmu should also be framed more cautiously. BioEmu is designed to sample a Boltzmann distribution, whereas AlphaFold models do not explicitly aim to reproduce thermodynamic ensembles. Therefore, direct comparison on the task of recovering experimental alternative conformations may not be fully fair without clarifying what is being measured: recall of known states versus sampling of physically meaningful distributions.

Response 4.4. Very fair point. We actually agree with the reviewer that this might be a general difference between models trained to reproduce distributions vs. identifying states (with different strengths), although we prefer to be a bit cautious and not conclusively claim so based on a single comparison. Also relevant to Responses 3.3 and 4.3, we have adjusted the Results accordingly (p. 5):

Notably, BioEmu is trained to perform Boltzmann sampling of states for proteins, rather than recovering experiment-like structures. Indeed, we found that BioEmu sampled significantly more diverse conformational ensembles compared to AF3 in terms of the Vendi score.

We have also edited the concluding paragraph of Discussion to emphasize this point (p. 14):

In conclusion, we have shown how several different MSA perturbations improve sampling of alternative states in AF3. Although these methods alone do not capture the entire underlying Boltzmann distribution, they provide valuable structural hypotheses, and were not improved on by the BioEmu model specifically trained to generate Boltzmann distributions of conformations.

4.5. Finally, the dataset requires clearer description: are alternative conformations observed under comparable experimental conditions, or are they driven by binding partners, ligands, or environmental changes (pH, ionic strength, etc.) that are not represented in the AF3 inputs? This could strongly affect interpretation, and might explain cases where BioEmu (and also AF) fails to sample certain states that are very unlikely in the absence of an interacting molecule, for instance.

Response 4.5. We agree that clearer dataset descriptions can improve the interpretability of our results, and have adjusted Methods to expand on this point (p. 14):

The dataset includes both proteins where the experimental structures were solved under comparable conditions and proteins where the distinct conformations were determined with and without binding partners, and/or under different environmental conditions.

Thank you for the thoughtful consideration of our manuscript (COMMSCHEM-26-0288). As demonstrated in the attached revision and outlined below, we believe we have responded to all concerns of reviewers 1–4.

In this response, reviewer comments are gray.

Reviewer #1: I think the authors have done a good job of responding to the points raised by the reviewers, and I recommend publication.

Response 1. We thank the reviewer for their comments.

Reviewer #2: I co-reviewed this manuscript with one of the reviewers who provided the listed reports. This is part of the Communications Chemistry initiative to facilitate training in peer review and to provide appropriate recognition for Early Career Researchers who co-review manuscripts.

Response 2. We thank the reviewer for their time and attention.

Reviewer #3: The authors have adequately addressed the concerns raised. The revisions and clarifications provided have significantly improved the clarity of the manuscript, better defined its scope, and strengthened the presentation and impact of the work.

Response 3. We thank the reviewer for their comments.

Reviewer #4: I thank the authors for their careful revision of the manuscript and for providing detailed responses to the reviewers' comments. I appreciate the substantial effort invested in improving the work. In particular, the additional analyses of sampling diversity and the expanded discussion have strengthened the manuscript.

That said, my main concerns are only partially addressed.

My original review emphasized that the study is primarily descriptive and that its scientific impact would be significantly enhanced by a stronger mechanistic understanding of why the proposed perturbation strategies improve conformational sampling in AF3. The additional discussion regarding the effects of amino acid masking is welcome, but it remains largely speculative. Likewise, the discussion of the diffusion model highlights interesting future directions but does not substantially clarify the origin of the observed improvements.

Consequently, I continue to view this work as an incremental extension of previous studies on MSA perturbation strategies rather than a fundamentally new methodological advance. Nevertheless, I acknowledge that the benchmark is extensive, the analyses are carefully conducted, and the results should be valuable to researchers interested in AF3 conformational sampling.

Response 4. We are glad that the reviewer appreciates our revisions. While we humbly acknowledge that our results can be seen as extensions of previous studies rather than fundamentally new advances, we appreciate that the reviewer finds the results to be valuable for other researchers.
