# Supplementary material for: Several multiple sequence alignment-perturbing methods enhance AlphaFold3 sampling of alternative protein states
Source: Commun Chem. 2026 Sep 18;9:300. doi: 10.1038/s42004-026-02198-x (PMC13588791; doi:10.1038/s42004-026-02198-x)
Supplement: Supplementary file 2 [file 42004_2026_2198_MOESM2_ESM.pdf]

Supplementary Information

Several multiple sequence alignment-perturbing methods enhance AlphaFold3 sampling of alternative protein states

Samuel Eriksson Lidbrink[1], Ivan Nissen[2†],
Jonathan Kenichi Ahrlind[2†], Rebecca J Howard[1,2],
Erik Lindahl[1,2,3,4*]

[1]Science for Life Laboratory, Department of Biochemistry and Biophysics, Stockholm University, Solna, Sweden.
[2]Science for Life Laboratory, Department of Applied Physics, KTH Royal Institute of Technology, Solna, Sweden.
[3]Department of Physics, Chemistry and Biology, Linköping University, Linköping, Sweden.
[4]Department of Chemistry, University of Illinois at Urbana-Champaign, Urbana, IL, United States.

*Corresponding author(s). E-mail(s): erik.lindahl@dbb.su.se;
Contributing authors: samuel.eriksson@scilifelab.se;
rebecca.howard@dbb.su.se;
†These authors contributed equally to this work.

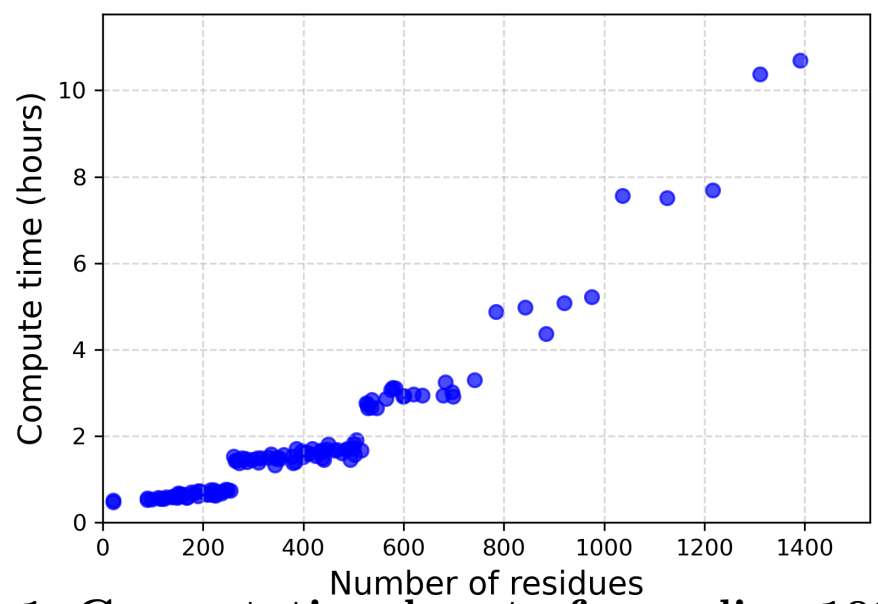

Supplementary Figure 1 Computational cost of sampling 1000 AlphaFold3 predictions (excluding MSA generation). The sampling was performed on an NVIDIA A100-SXM4-40GB GPU.

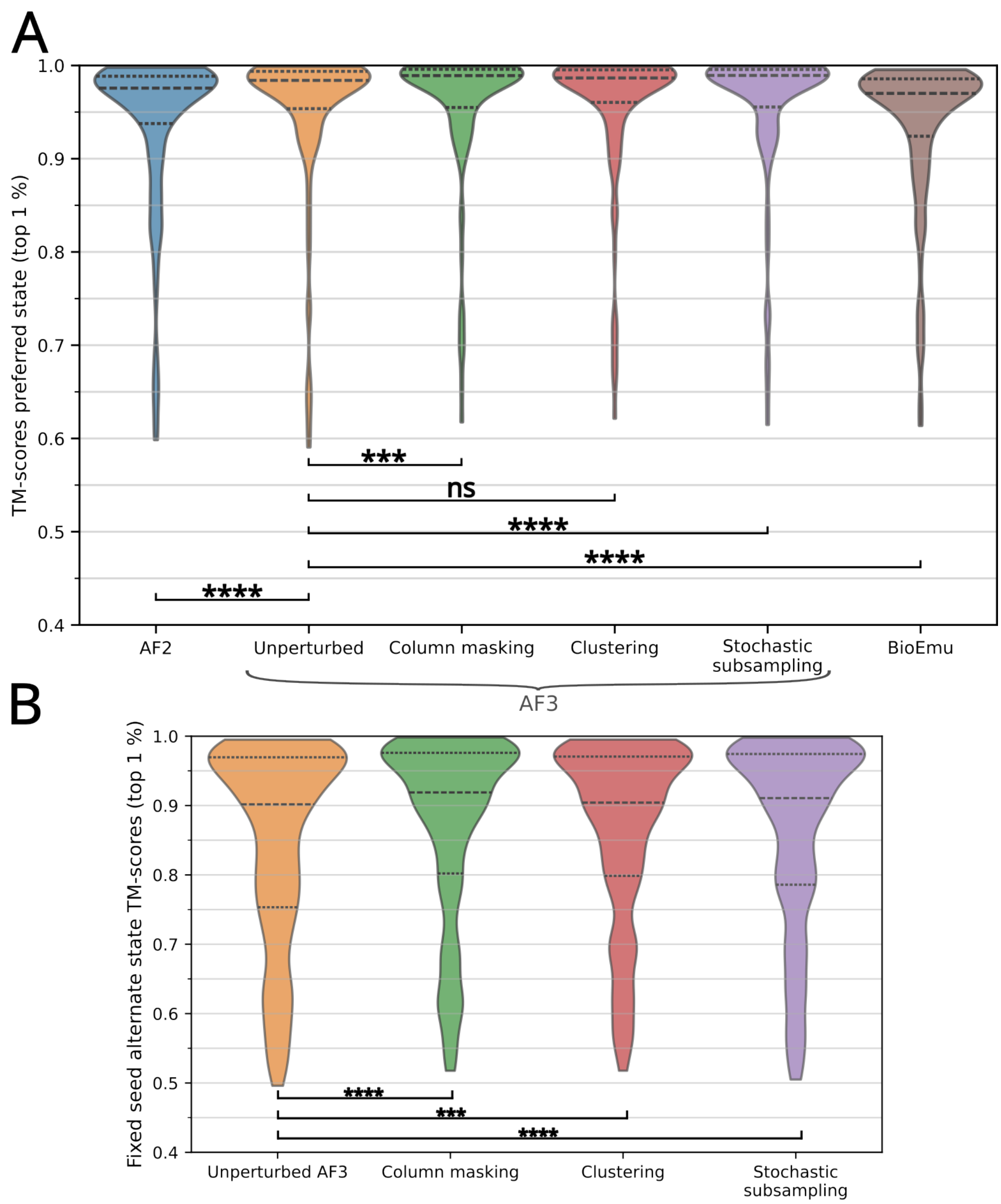

Supplementary Figure 2 Additional TM-score distributions. Violin plots showing the distributions of the mean top 1% TM-scores of (A) the preferred state for all methods, and (B) the alternative state per latent space in AF3 (i.e. per fixed seed), both across the entire dataset of 107 proteins. Statistical significance is assessed using the two-sided Wilcoxon signed-rank test (*: $p < 0.05$, **: $p < 0.01$, ***: $p < 0.001$, ****: $p < 0.0001$). Dashed and dotted lines indicate median and quartile values, respectively.

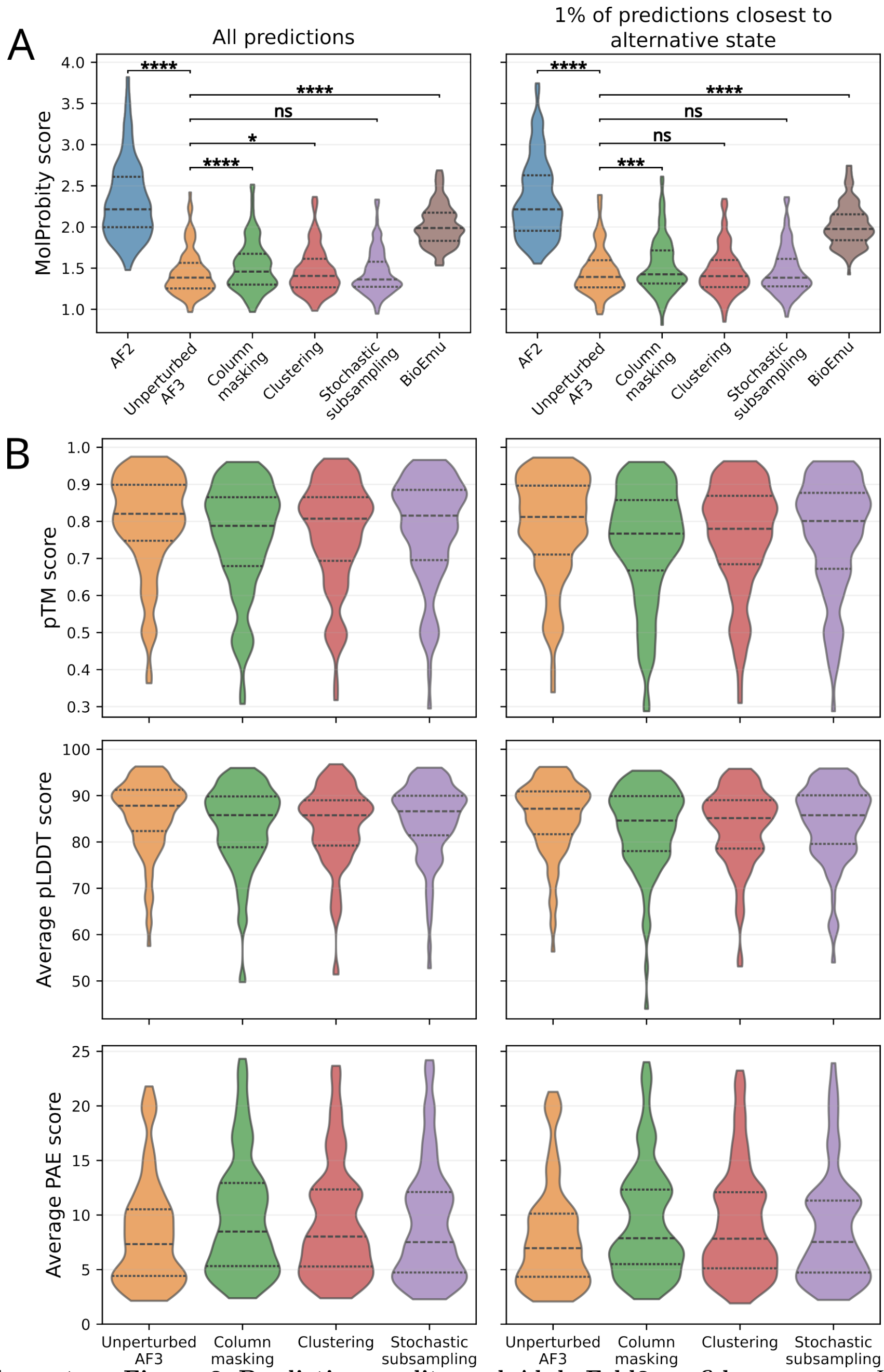

Supplementary Figure 3 Prediction quality and AlphaFold3 confidence scores. Violin plots showing the distributions across the examined proteins of (A) the MolProbity scores and (B) the pTM, average pLDDT, and average PAE scores, averaged over all predictions per protein (left) and averaged over the 1% of predictions that were closest to the alternative state (right). Statistical significance is assessed using the two-sided Wilcoxon signed-rank test (*: $p < 0.05$, **: $p < 0.01$, ***: $p < 0.001$, ****: $p < 0.0001$). All AF3 confidence scores differ significantly between unperturbed AF3 and all perturbation methods, with p-values all below 0.0001. Dashed and dotted lines indicate median and quartile values, respectively.

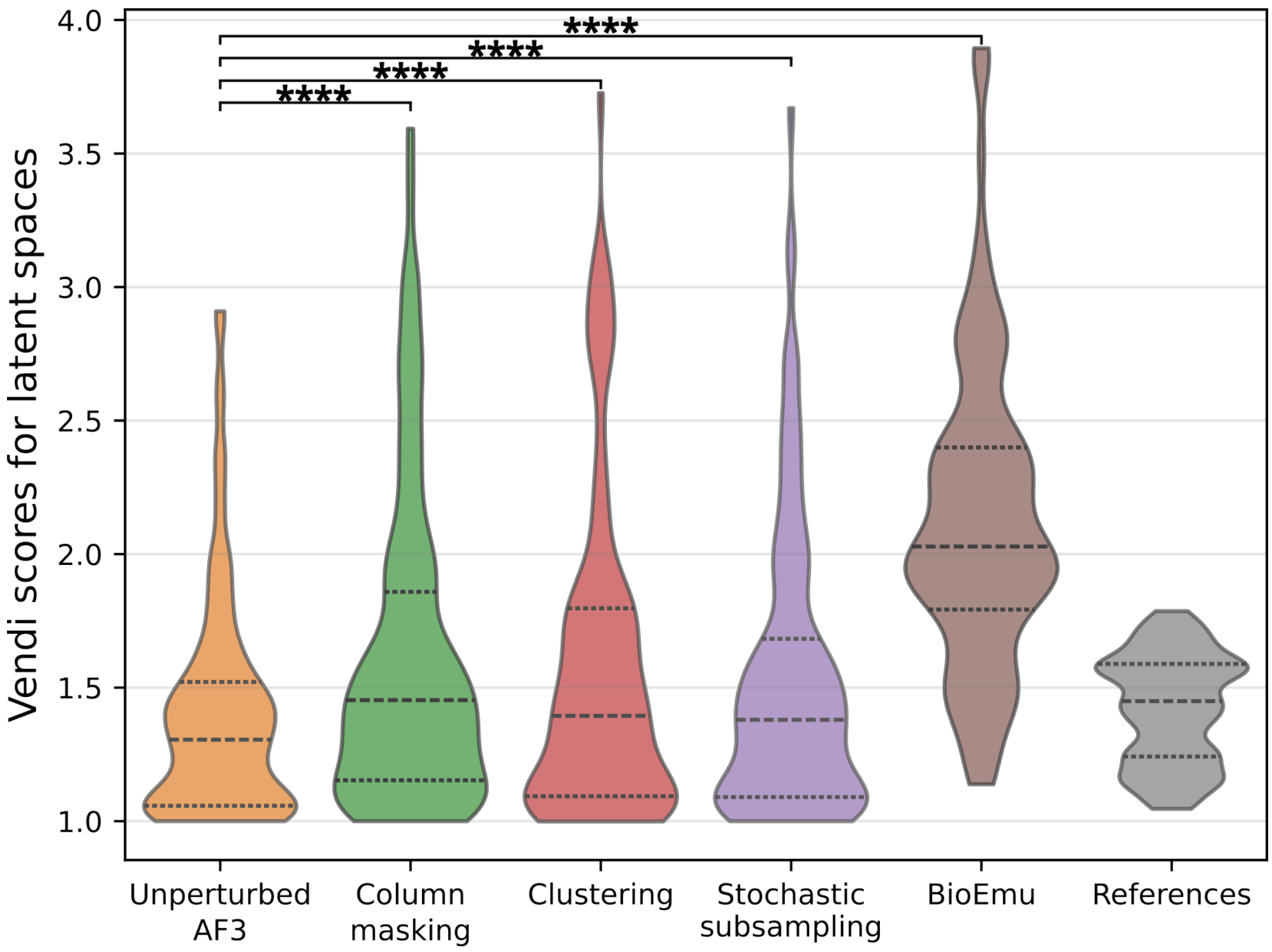

Supplementary Figure 4 BioEmu and MSA-perturbed AlphaFold3 produce more structurally diverse latent spaces compared to unperturbed AlphaFold3. Violin plots showing the distribution over the examined proteins of the maximum Vendi score per latent space, where each latent space contains a maximum of five structure predictions for consistency. For BioEmu, this meant randomly partitioning the total latent space of 1000 predictions into 200 sub-spaces, each with five predictions. All predictions with MolProbity scores above 3.0 were filtered out for all methods, filtering 0.05% of the column-masked predictions, 0.2% of the BioEmu predictions, and no predictions for the remaining methods. The distribution of Vendi scores for two-state models with both experimental reference structures per protein is shown in gray. Statistical significance is assessed using the two-sided Wilcoxon signed-rank test (*: $p < 0.05$, **: $p < 0.01$, ***: $p < 0.001$, ****: $p < 0.0001$). Dashed and dotted lines indicate median and quartile values, respectively.

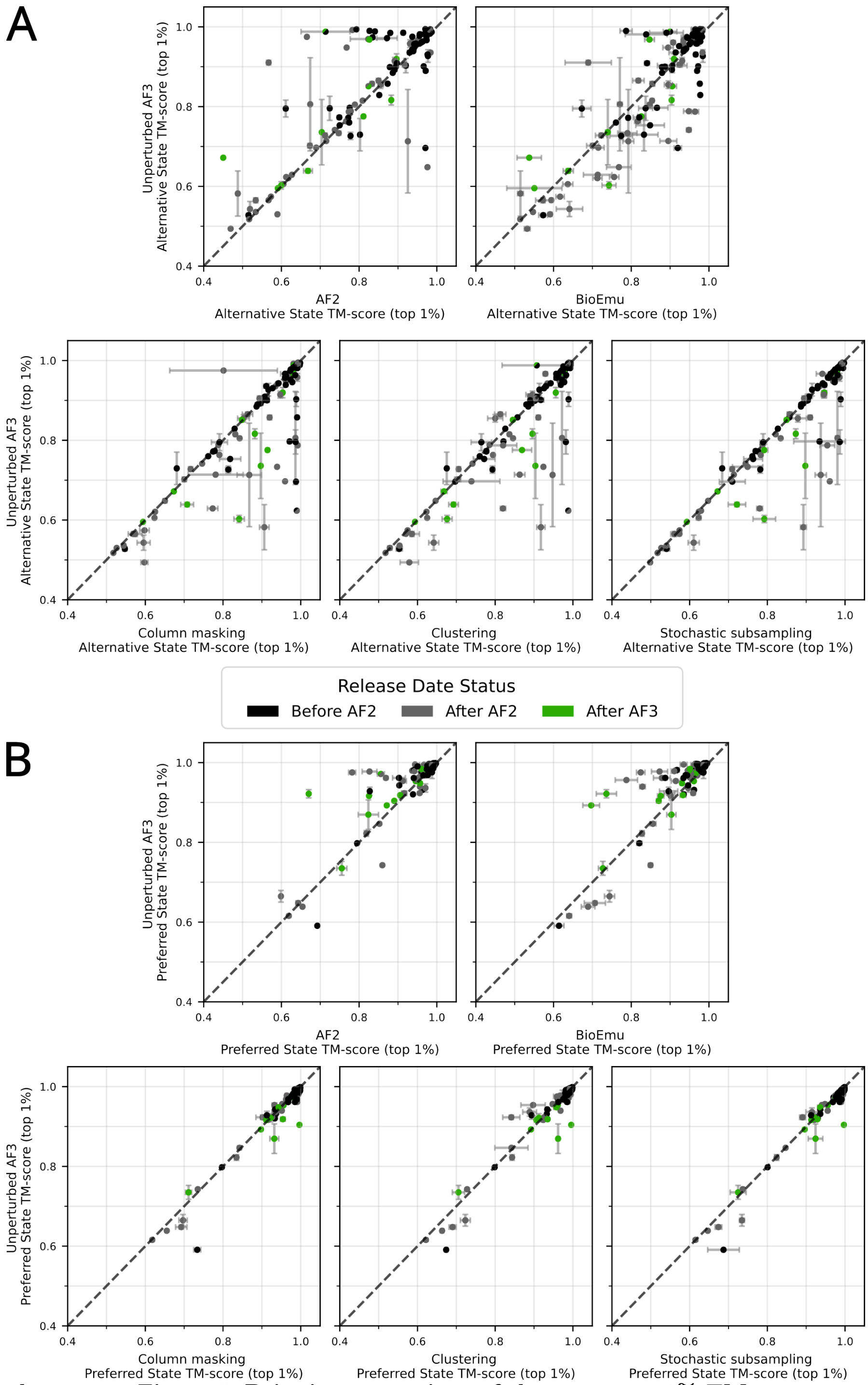

Supplementary Figure 5 Pairwise comparison of the mean top 1% TM-scores per protein between unperturbed AF3 and the remaining methods. Shown for (A) the alternative state and (B) the preferred state. Error bars indicate the standard deviation of the corresponding top 1% TM-scores.

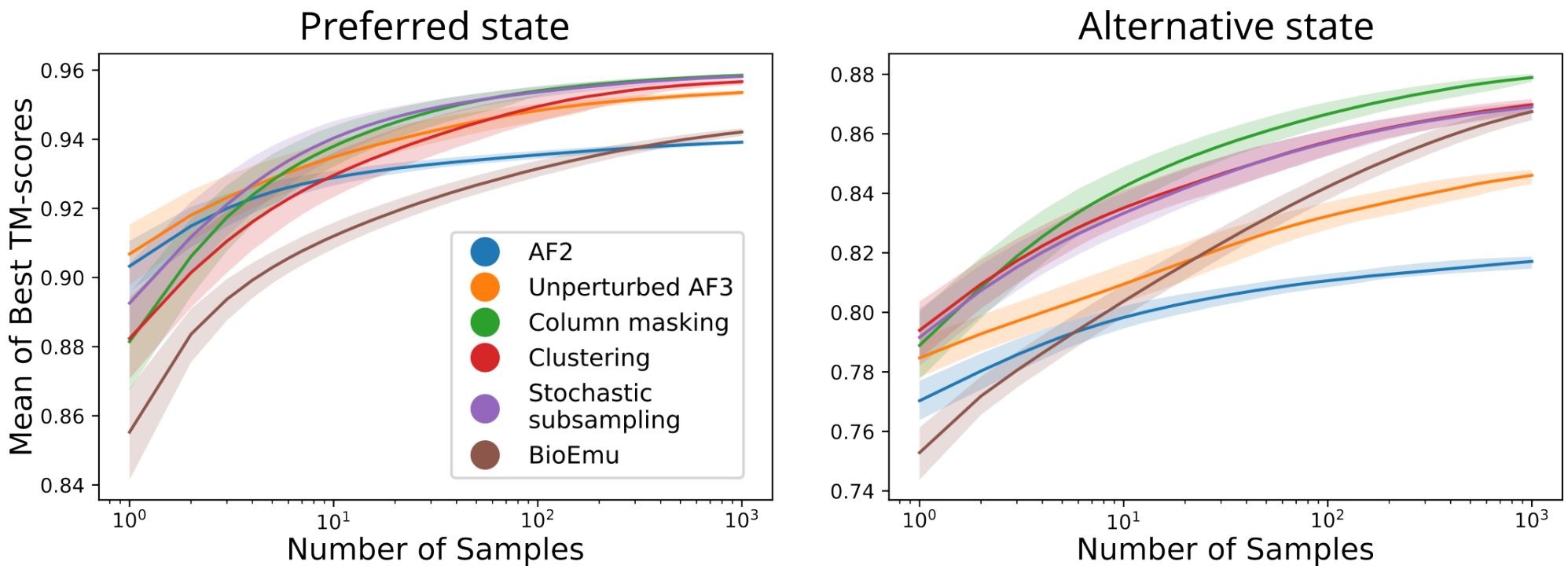

Supplementary Figure 6 The effect of increased sampling varies across different sampling methods. Shaded regions indicate 95% confidence intervals estimated by bootstrapping the sampled conformations, reflecting the inherent sampling uncertainty.

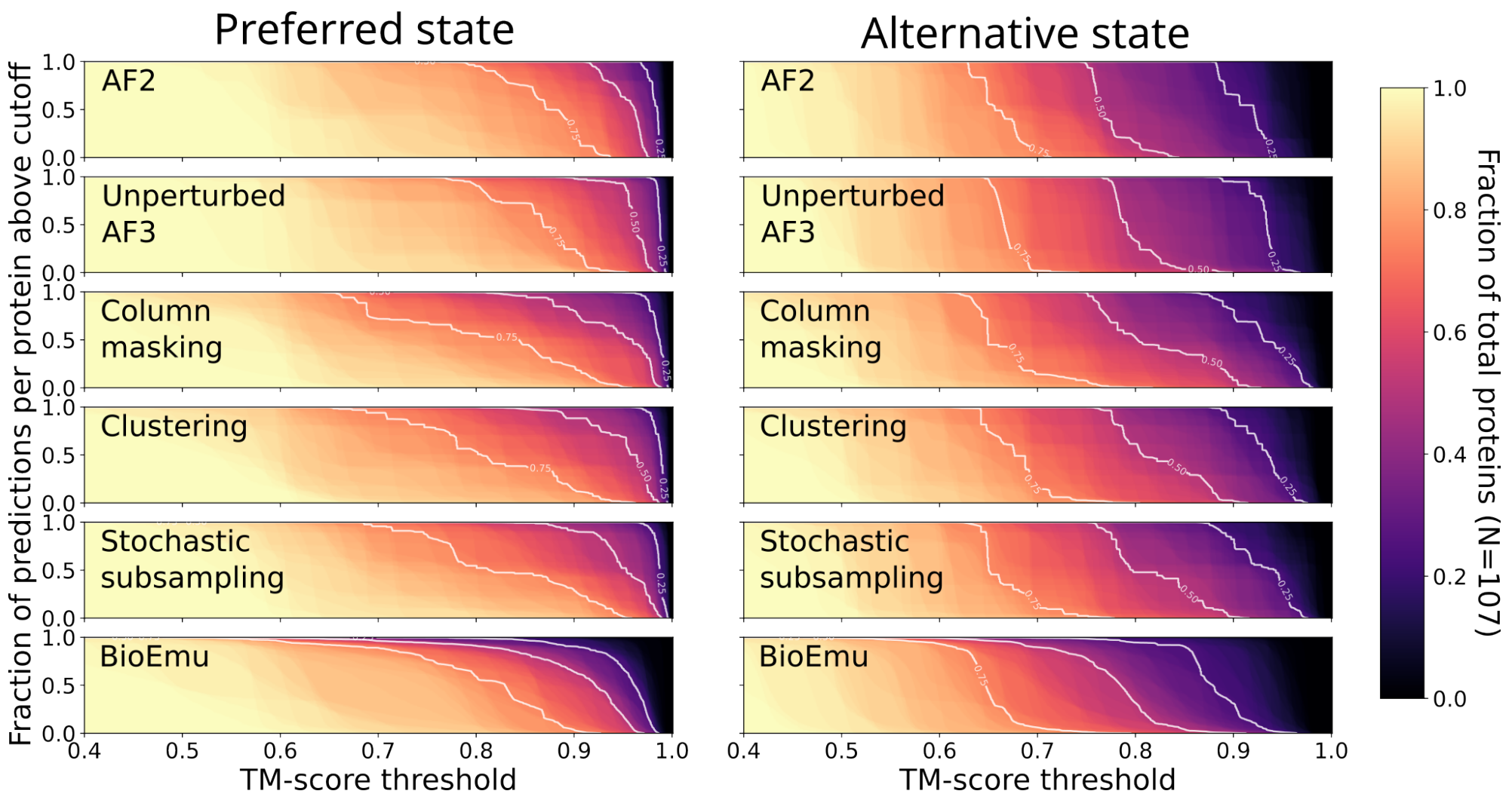

Supplementary Figure 7 MSA perturbations increase probabilities of sampling alternative states in AF3. Heatmap showing what fractions of the predictions per protein (y-axis) have TM-scores above different thresholds (x-axis). Brighter hues indicate that more proteins in the dataset sample at least the given fraction of predictions with TM-scores above the given threshold. The TM-scores are calculated with respect to the preferred state (left) and alternative state (right). The white lines indicate the isolines for 75%, 50% and, 25% of the total number of proteins.

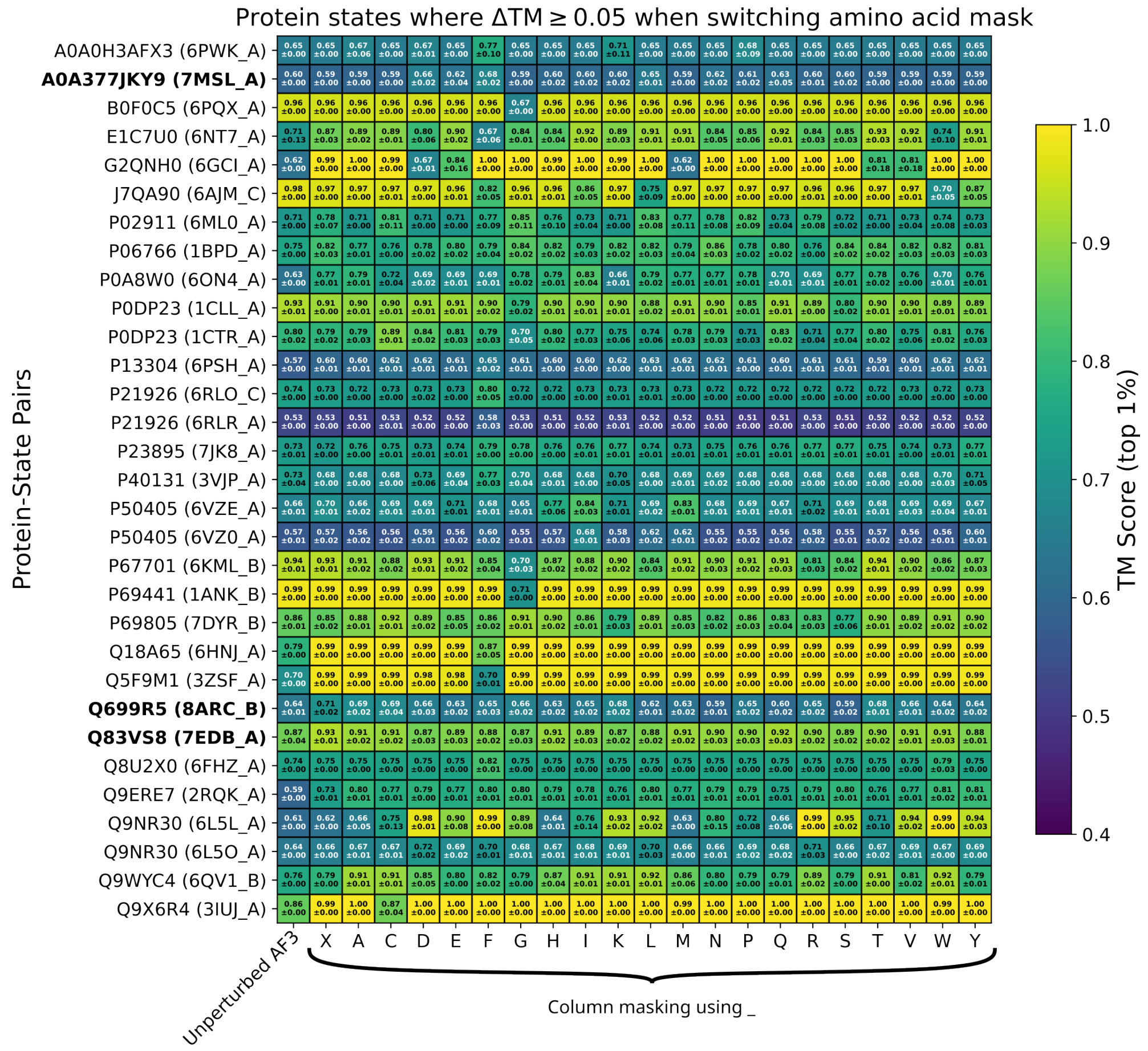

Supplementary Figure 8 The optimal choice of amino acid column mask varies for different proteins. Heatmap of the top 1% TM-scores (mean ± standard deviation) for either unperturbed AF3 or column-masked AF3 with a given mask, for the 31 out of 110 protein-state pairs (representing both states across the 55 evaluated proteins) where the mean top 1% TM-score differed by $\Delta\mathrm{TM} \geq 0.05$ when switching from X to any other amino acid mask. Reference structures that were released after the AF3 training date cutoff are shown in bold.

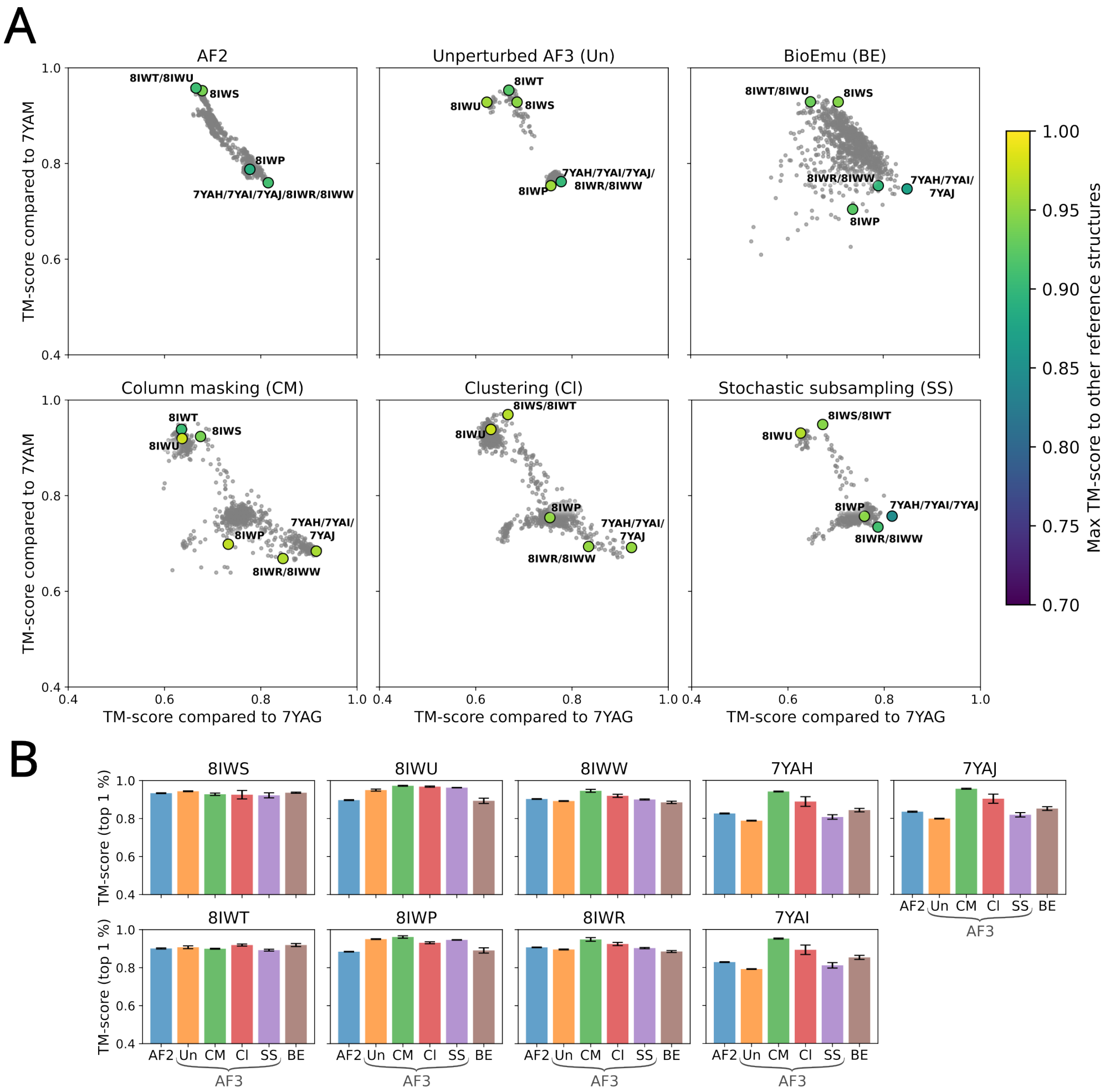

Supplementary Figure 9 Structure predictions of the Secretory pathway Ca^{2+}-transporting ATPase type 1 for all methods and all PDB-deposited reference states. (A) Diversity plots showing the TM-scores of all predictions to reference structures in the E1-ATP state (PDB ID: 7YAG) and the E2P state (PDB ID: 7YAM). Colored points mark the structure predictions that are closest to another reference structure in the PDB. The 8IWP reference structure represents an ion-bound (CaE1) state. (B) Mean top 1% TM-scores for each method compared to all additional PDB-deposited structures. Error bars indicate the standard deviation of the top 1% TM-scores (with $n = 37$ for clustering and $n = 10$ for the remaining methods).

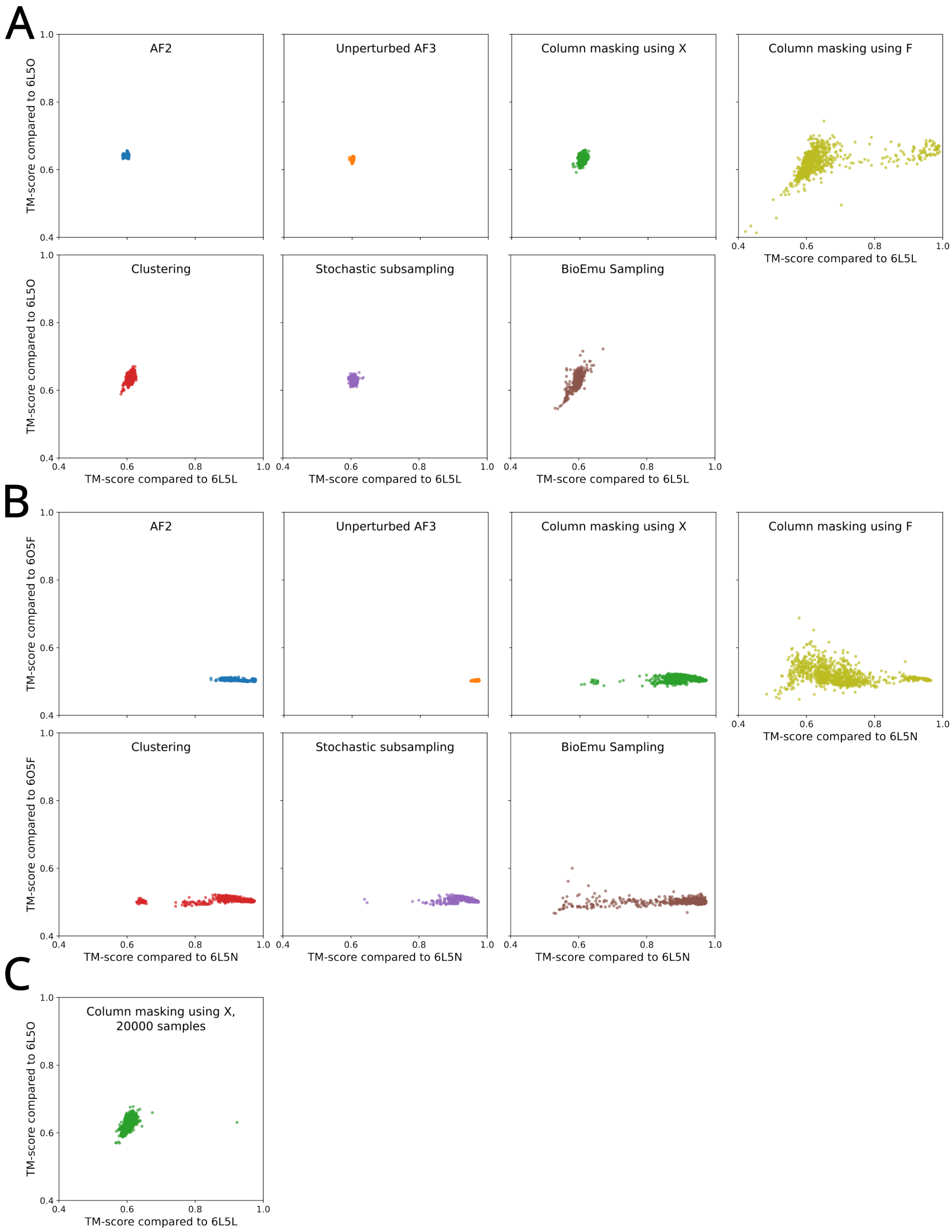

Supplementary Figure 10 Structure predictions of the Nucleolar RNA helicase 2 for all methods and all PDB-deposited reference states. Diversity plots showing the TM-scores for all predictions to experimental reference structures. Predictions are shown from (A, B) all methods, and (C) an expanded rerun of column masking. The reference structures corresponds to (A, C) the apo state (PDB ID: 6L5L) and the post-hydrolysis state (PDB ID: 6L5O), and (B) the post-unwound/pre-hydrolysis state (PDB ID: 6L5N) and the pre-unwound state (PDB ID: 6O5F).

Supplementary Table 1 Proteins and reference structures used in the benchmark.

UniProt ID	PDB ID 1	PDB ID 2
A0A075Q0W3	6MKA_A	6MKJ_A
A0A0H3AFX3	6PWJ_A	6PWK_A
A0A377JKY9	7F7P_A	7MSL_A
A0QTT2	7CY2_A	7CYR_A
A2RJ53	3FTO_A	3DRF_A
A6UVT1	6HAC_A	6HAE_A
B0F0C5	6PQN_A	6PQX_A
B3EYN2	5HO2_A	5HO0_A
B7IE18	6NC7_A	6NC6_A
E1C7U0	6NT7_A	6NT6_A
F1NCD6	7MJS_X	7N98_A
G2QNH0	6GCI_A	4C9J_B
J7QA90	6GTS_B	6AJM_C
J7QAK3	6E9N_B	6E9O_B
K0JNC6	7OZ3_A	7B5Y_A
M1GSK9	6P3X_A	7UU3_A
M1VAN7	6A6N_A	7DQV_A
O74933	2YQC_A	2YQS_A
O76728	4BP8_A	4BP9_A
O86150	5C78_A	6HRC_D
P00558	2XE6_A	2WZD_A
P00766	2CGA_B	1AFQ_C
P02787	1BP5_A	1RYO_A
P02911	6ML0_A	6MLP_A
P02925	1URP_A	2DRI_A
P03047	7UBJ_A	7UBL_A
P03960	7LC3_B	7BGY_B
P06766	1BPD_A	2BPG_E
P08037	1PZT_A	1PZY_D
P08709	1JBU_A	1WUN_B
P0A7D4	1ADE_A	1CIB_A
P0A7F3	2AIR_D	1ZA1_D
P0A8V6	1E2X_A	1H9G_A
P0A8W0	6ON4_A	6WFQ_A
P0AEQ3	1WDN_A	1GGG_A
P0AEX9	3PUW_A	1FQC_A
P0AEY8	6VS1_A	6GV1_A
P0AG16	1ECJ_D	1ECC_B
P0CL43	6XFJ_A	6XFL_A
P0DP23	1CLL_A	1CTR_A
P12758	1K3F_B	1U1D_F
P13304	6PX4_A	6PSH_A
P18238	6GCI_A	4C9J_B
P19491	1MY1_C	1FTL_A
P20789	6Z4Q_A	6Z8N_A
P21589	7QGA_A	4H2I_A
P21926	6RLO_C	6RLR_A
P23847	1DPE_A	1DPP_A
P23895	7MGX_A	7JK8_A
P24182	1BNC_B	2V5A_A
P26281	1HKA_A	3IP0_A
P28366	1M74_A	1TF2_A
P30878	7L17_A	4M64_A
P31133	6YED_A	6YE0_A

UniProt ID	PDB ID 1	PDB ID 2
P33284	3O80_A	3O8M_A
P37487	1K23_C	1WPM_B
P40131	3TEE_A	3VJP_A
P43005	8CTC_A	6S3Q_A
P44542	2CEY_A	6H76_A
P45568	1K5H_C	2EGH_B
P50405	6VZE_A	6VZ0_A
P54708	8IJL_A	7X23_A
P56817	1W50_A	3IXJ_C
P60338	1EXM_A	1HA3_B
P60752	7SEL_A	8DMM_A
P61586	1XCG_B	1OW3_B
P62495	2KTV_A	2KTU_A
P62593	1JWP_A	1PZO_A
P67701	6JQ4_A	6KML_B
P68082	7DGJ_A	6LS8_A
P69441	4AKE_B	1ANK_B
P69801	7DYR_A	6K1H_B
P69805	7DYR_B	6K1H_A
P71447	2WFA_A	2WF5_A
P79345	1NEP_A	2HKA_C
P84080	1RRG_A	1S9D_A
P98194	7YAG_A	7YAM_A
P9WPY3	2IYT_A	2IYQ_A
Q02153	6JT0_B	7D9R_B
Q02763	1FVR_A	2OO8_A
Q16539	2ZB1_A	3HL7_A
Q16658	3P53_A	6I11_A
Q18A65	6HNJ_A	6HNI_A
Q27686	1PKL_B	3HQP_P
Q29495	1B6B_A	1KUV_A
Q2FYI5	8E2C_A	8E2B_A
Q31PX7	7N82_A	6UF2_A
Q53W80	7C63_A	7C66_A
Q58L87	2WGB_A	2V57_A
Q5F9M1	3ZSF_A	2YLN_A
Q63GK8	6BVG_A	5IWS_A
Q699R5	8ARC_B	8ARB_B
Q6MLJ0	6BTX_A	5AYM_A
Q72J05	5MKK_B	6RAJ_B
Q7DAU8	3L6G_A	3L6H_A
Q83VS8	7E8R_A	7EDB_A
Q8U2X0	6FHZ_A	3VVS_A
Q8WSF8	3PEO_G	2BYS_J
Q9DA75	7MJS_X	7N98_A
Q9ERE7	2RQM_A	2RQK_A
Q9NR30	6L5L_A	6L5O_A
Q9SS90	6K8B_A	6K85_B
Q9WYC3	3QF4_A	6QV1_C
Q9WYC4	4Q4A_B	6QV1_B
Q9X6R4	3IUJ_A	3IUQ_A
Q9X9P9	2OLO_A	2OLN_A
Q9Z4N6	1SI1_A	1SI0_A

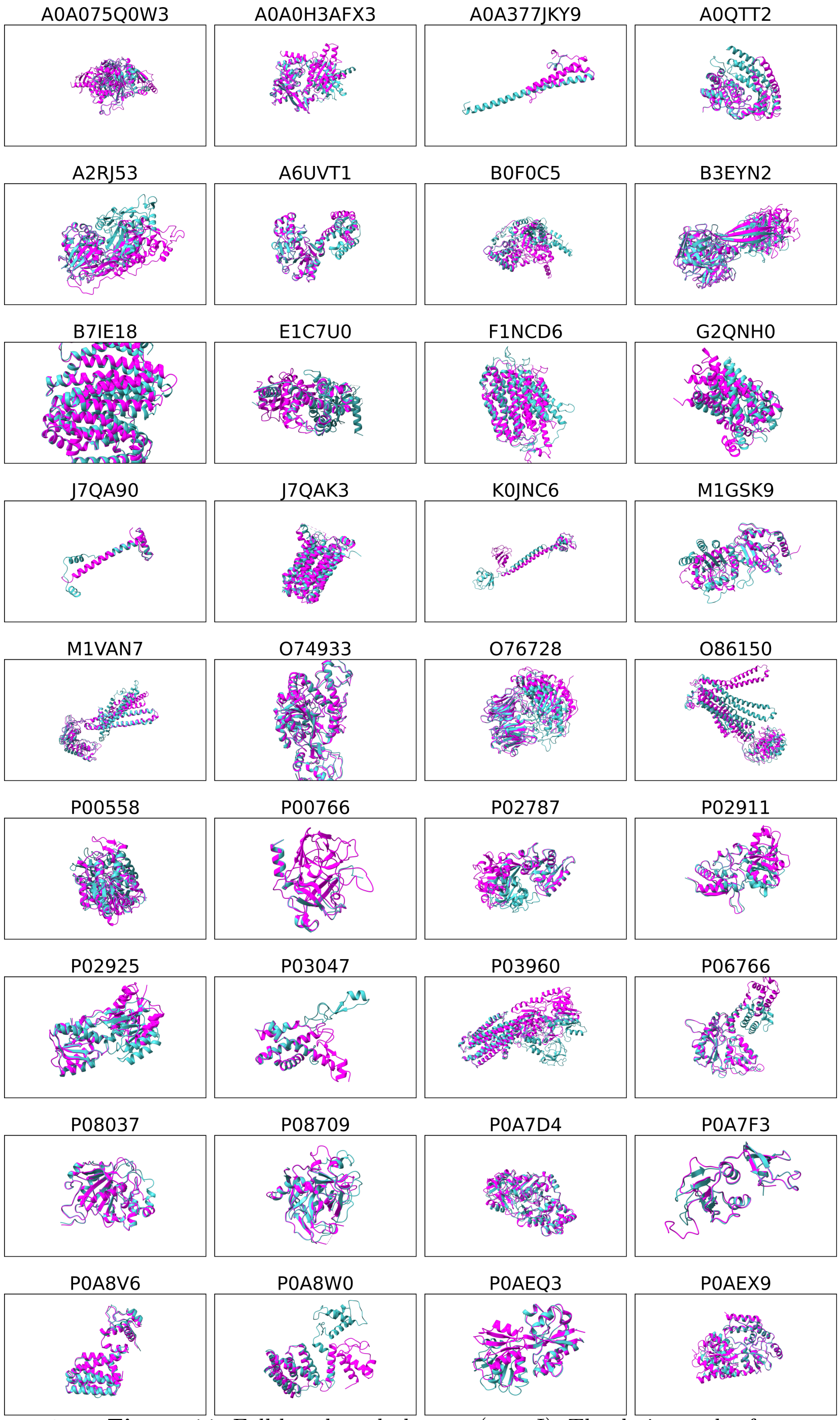

Supplementary Figure 11 Full benchmark dataset (part I). The designated reference states are visualized for each UniProt ID using ChimeraX [1].

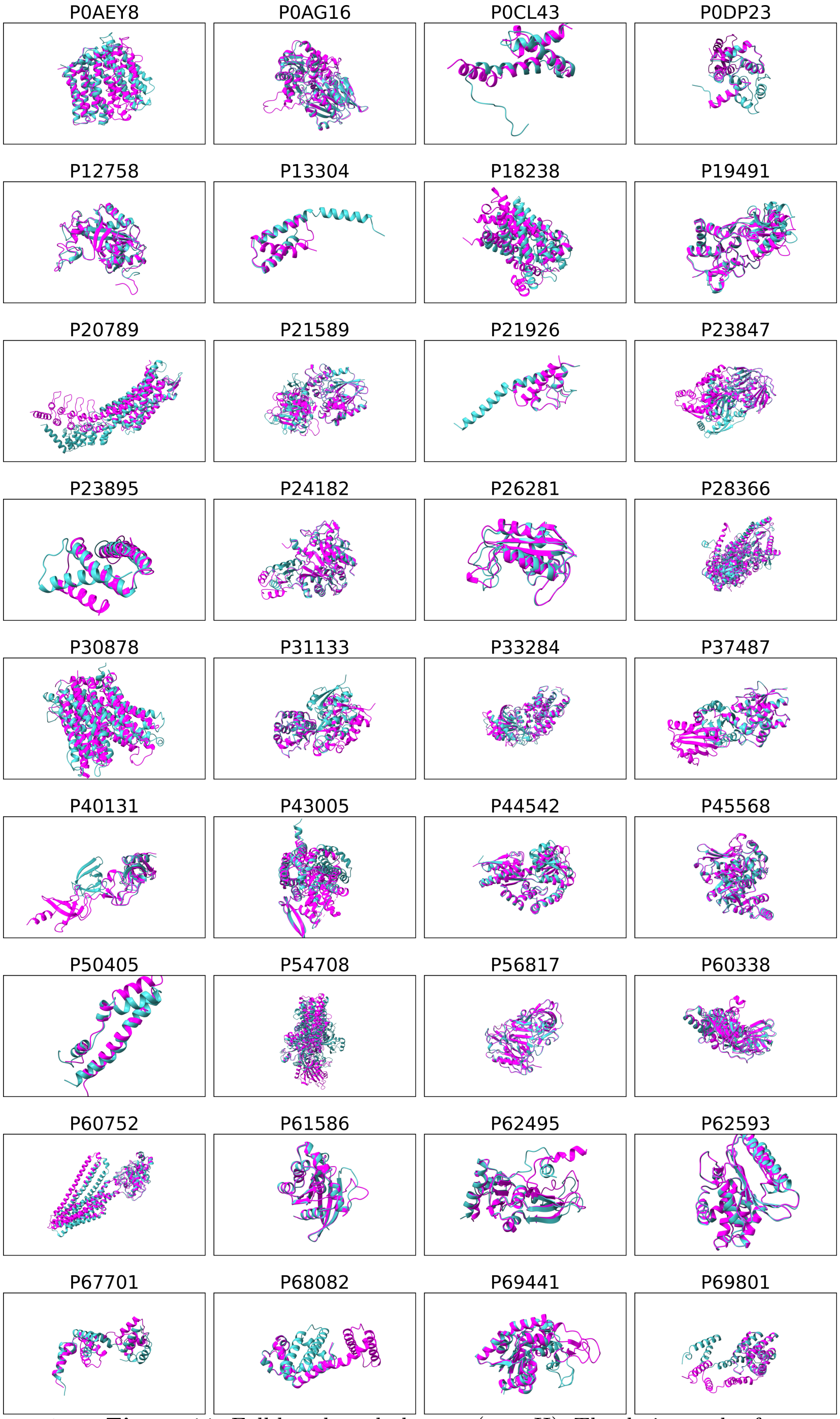

Supplementary Figure 11 Full benchmark dataset (part II). The designated reference states are visualized for each UniProt ID using ChimeraX [1].

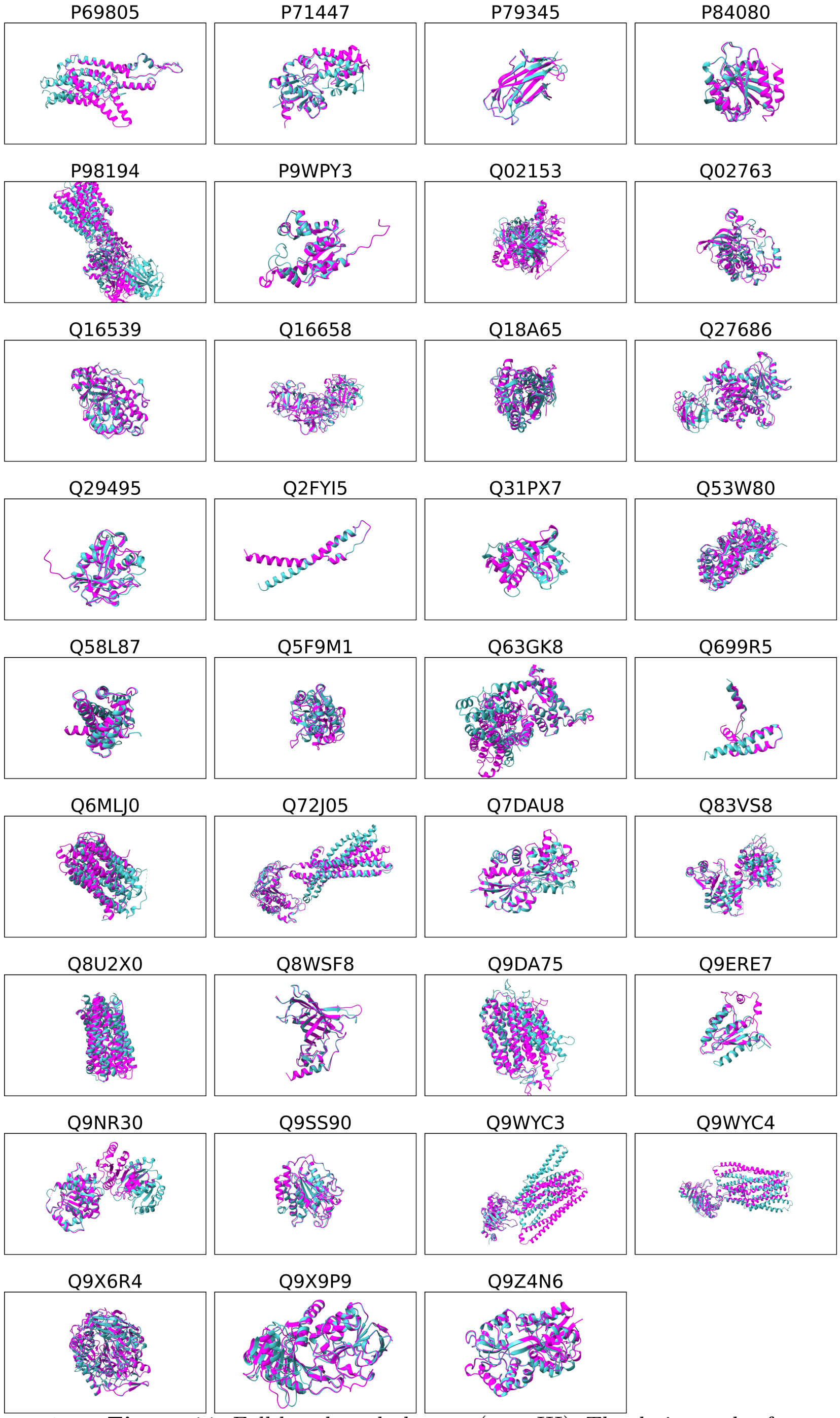

Supplementary Figure 11 Full benchmark dataset (part III). The designated reference states are visualized for each UniProt ID using ChimeraX [1].

References

[1] Meng, E. C. *et al.* Ucsf chimerax: Tools for structure building and analysis. *Protein Science* **32**, e4792 (2023). URL https://onlinelibrary.wiley.com/doi/abs/10.1002/pro.4792.
